# miR-126 inhibits papillary thyroid carcinoma growth by targeting LRP6

**DOI:** 10.3892/or.2021.8097

**Published:** 2021-05-31

**Authors:** Qiang Wen, Jie Zhao, Lin Bai, Tongtong Wang, Haishan Zhang, Qingjie Ma

Oncol Rep 34: 2202-2210, 2015; DOI: 10.3892/or.2015.4165

Following the publication of this paper, it was drawn to the Editors attention by a concerned reader that certain of the western blotting data shown in Figs. 4C, 5B and 6D were strikingly similar to data appearing in different form in other articles by different authors. Owing to the fact that the contentious data in the above article were already under consideration for publication, or had already been published, elsewhere prior to its submission to *Oncology Reports*, the Editor has decided that this paper should be retracted from the Journal. After having been in contact with the authors, they agreed with the decision to retract the paper. The Editor apologizes to the readership for any inconvenience caused.

